# Associations Between Sleep Duration and Burnout Dimensions Among Healthcare Workers in a Public Hospital in Pakistan

**DOI:** 10.1192/bjo.2026.11207

**Published:** 2026-06-30

**Authors:** Minnahil Ali, Shahab Muhammad, Zainab Farooq

**Affiliations:** 1 HSE North Dublin Mental Health Services, Dublin, Ireland; 2 Pak Naval Services Shifa Hospital, Karachi, Pakistan; 3 HSE North Dublin City Mental Health Service, Dublin, Ireland

## Abstract

**Aims:**

Burnout among healthcare workers is an increasing mental health concern, particularly in low- and middle-income countries such as Pakistan, where public-sector hospitals operate under substantial workforce and resource constraints. Burnout is conceptualized as a multidimensional psychological response to chronic occupational stress, encompassing emotional exhaustion, depersonalization, and reduced personal accomplishment. This study hypothesized that shorter sleep duration would be associated with higher levels of burnout across these dimensions.

**Methods:**

A cross-sectional correlational study was conducted among healthcare workers employed at a public-sector hospital in Pakistan. Participants included doctors, nurses, and allied healthcare professionals across medical and surgical specialties (N=92–93). Average daily sleep duration was self-reported. Burnout was assessed using the Maslach Burnout Inventory (MBI), measuring emotional exhaustion, depersonalization, and personal accomplishment. Pearson product–moment correlation analyses were performed to examine associations between sleep duration and burnout dimensions, as well as interrelationships among burnout subscales.

**Results:**

Sleep duration was not significantly associated with emotional exhaustion (r=−0.12, p=0.26), depersonalisation (r=−0.08, p=0.46), or personal accomplishment (r=0.10, p=0.35). A moderate positive correlation was observed between emotional exhaustion and depersonalisation (r=0.39, p < 0.01), indicating that higher exhaustion levels were associated with greater emotional distancing. No statistically significant associations were found between personal accomplishment and the other burnout dimensions.

**Conclusion:**

Although sleep duration alone did not demonstrate a significant relationship with burnout, these findings contribute to an understanding of burnout as a multi factorial psychological phenomenon. The strong association between exhaustion and depersonalization reflects a core burnout pathway involving emotional depletion and disengagement. From a mental health standpoint, the results highlight the importance of addressing systemic and workplace-related stressors through mental health-informed interventions in Pakistani public hospitals, rather than focusing solely on individual lifestyle factors.

